# Hypertension and prehypertension among adolescents attending secondary schools in urban area of South-East, Nigeria

**DOI:** 10.11604/pamj.2018.31.145.15994

**Published:** 2018-10-25

**Authors:** Chijioke Elias Ezeudu, John Onuora Chukwuka, Joy Chinelo Ebenebe, Wilson Chukwuneke Igwe, Ifeoma Egbuonu

**Affiliations:** 1 Department of Paediatrics, Nnamdi Azikiwe University, Awka, Nnewi Campus, Nigeria; 2 Department of Paediatrics, Chukwuemeka Odimegwu Ojukwu University Teaching Hospital, Awka, Anambra State, Nigeria

**Keywords:** Hypertension, prehypertension, adolescent, secondary school

## Abstract

**Introduction:**

in the past, the need for regular blood pressure screening in children was doubtful, and the main reason against it is that hypertension is an adult illness and there is no evidence that screening healthy children for hypertension was worthwhile. We did this study to determine the prevalence of hypertension and prehypertension as well as some risk factors for hypertension among secondary school adolescents in an urban area of the South-East, Nigeria.

**Methods:**

this was a cross-sectional study of 984 adolescents aged 10-19 years in secondary schools in Awka South Local Government Area of Anambra state, South-East, Nigeria. The multi-stage sampling method was used to select the subjects. Data were collected from all eligible subjects with the aid of a questionnaire administered to them. Weight, height, and blood pressure were measured and recorded.

**Results:**

nine hundred and eighty-four adolescents were recruited for this study, and they comprised 470 (47.8%) males and 514 (52.2%) giving a male: female ratio of 1:1.1. Their ages ranged from 10-19 years. The mean systolic blood pressure and mean diastolic BP were 110.5±10.2mmHg 71.5±8.5mmHg respectively. Prevalence of hypertension and pre-hypertension were 6.3% and 5.0% respectively. There were a higher proportion of females (7.3%) than males (5.4%) with hypertension, and more females (5.8%) than males (4.2%) with prehypertension but these were not statistically significant. Overweight and obesity were significantly associated with hypertension.

**Conclusion:**

hypertension exists among secondary school adolescents in Awka South Local government area of Anambra state, with a prevalence of 6.3%. Early detection and treatment will forestall the early development of complications.

## Introduction

The value of blood pressure measurement as a screening tool for hypertension in adults has long been documented. In the past the need for regular blood pressure screening in children was arguable and the main reason against it being that hypertension is an adult illness and there is no evidence that screening healthy children for hypertension was worthwhile [1]. Presently it has been established as an important component of routine physical examination among the paediatric population and should be carried out annually after three years or earlier in children with a history of neonatal problems, renal disease or familial risk factors [2]. Hypertension is an important public health problem affecting both adults and children. In children, hypertension is defined as systolic or diastolic blood pressure that is above the 95^th^ percentile for age, sex and height, while pre-hypertension is between 90^th^ and 95^th^ percentile [3]. High blood pressure and its complications are the leading cause of death in the world [4], and about one billion adults around the world had hypertension in the year 2000, and this number is expected to rise to 1.56 billion by 2025 [5]. Cardiovascular disease (CVD) is increasingly recognized as a leading cause of morbidity and mortality in Sub-Sahara Africa including Nigeria [6, 7]. It is increasingly evident that the origin of essential hypertension in adolescents may be traced to early life [8, 9]. In addition, the recognition that blood pressure elevation in childhood is a predictor of high blood pressure in adults, have led to renewed curiosity in investigating blood pressure and its correlates in childhood and adolescence [10, 11]. In adolescents, hypertension is often undetected because they are generally healthy and seldom visit a physician unless there is an obvious illness. For this reason, routine measurement of blood pressure among children and adolescents is recommended [3]. Detecting adolescents with prehypertension and hypertension would aid early treatment. Studies in recent times have demonstrated that the level and trend in blood pressure vary from population to population [12, 13]. In fact, among children and adolescents, growth patterns, age and gender strongly influence blood pressure levels [13]. Equally, socio-demographic factors, overweight and obesity in children has been associated with high blood pressure [14, 15]. It is important to continually assess blood pressure among children, as doing so, will provide information for the formulation of health-care policy and prevention strategies especially in developing countries where data are scarce [4]. In South-East Nigeria, particularly in Anambra state, there is a paucity of data on childhood hypertension. This study sought to determine the prevalence of hypertension and prehypertension among adolescents attending secondary schools in Awka South Local Government Area of Anambra State, Nigeria. It also determined the relationship between some risk factors among these adolescents.

## Methods

This was a cross-sectional study of adolescents aged 10-19years in secondary schools in Awka South Local Government Area (ASLGA) of Anambra state, Nigeria. The study was carried out between June 2013 and April 2014. Ethical approval was obtained from the ethics committee of the Nnamdi Azikiwe University Teaching Hospital Nnewi, Anambra State. Approval was also obtained from the Anambra State Ministry of Education. Subjects were 1000 students, selected using a multi-stage sampling method. All the secondary schools were grouped into public and private secondary schools. Based on student population, a ratio of two public schools to one private school was selected. Within selected schools, students were stratified along age and gender (males and females) using the class register. Subjects were selected from each stratum by simple random sampling (balloting). The selected students and their parents gave written and oral consent before being enrolled as subjects in the study. All the subjects had their urine examined for protein and those with significant proteinuria were excluded from the study. Data were collected from all eligible subjects with the aid of a questionnaire administered to them. Weight, height and blood pressure were measured and recorded. Body mass index was calculated using the weight and the height as; Weight (kg)/Height (M^2^) [16]. Height was measured with the subject standing, the two legs together and fully extended, and the heels, buttocks, shoulder blades and occiput in firm contact with the measuring rule, and readings recorded to the nearest 0.5cm using a stadiometer [17, 18] (model RGZ-120). The weight was measured to the nearest 0.5kg with minimal clothing (with their school uniforms on and shoes removed.) using 'Health scale' weighing scale model RGZ-120. The blood pressure was measured after at least 5 minutes of rest in a seated position using Accoson sphygmomanometer model DEKAMET, MK.3 made in England. The measurement was taken in the morning hours between 8.30am and 12 noon before the break time. The measurement was done as recommended in the Seventh Report of the Joint National Committee on Prevention, Detection, Evaluation, and Treatment of High Blood Pressure (JNC-7) [19] with the subjects sitting quietly and the right arm on a table at the level of the heart. An appropriately sized cuff, covering about two-thirds of the upper arm with the lower border not less than 2.5 cm from the cubital fossa, was applied after restricting clothing had been removed. The manometer was at the level of the cuff. The brachial artery was palpated and its position noted. The cuff was then inflated to a pressure of 30 mmHg above the level at which the radial pulse was no longer palpable. The stethoscope was placed over the brachial artery in the cubital fossa and the pressure in the cuff was deflated at 2 mmHg every second until the first Korotkoff sound is heard. This was recorded as the systolic blood pressure (SBP). The pressure in the cuff was further lowered until the sounds disappear completely. This is the fifth Korotkoff sound, and the corresponding pressure recorded as the diastolic blood pressure (DBP). The blood pressure was measured twice at an interval of 1-2 minutes and the mean recorded. Normal blood pressure (NBP) was defined as systolic and diastolic blood pressure that is <90^th^ percentile for gender, age and height [3]. Prehypertension was defined as systolic and diastolic blood pressure ≥ 90th percentile, but < 95^th^ percentile for gender, age, and height [2]. Hypertension is defined as systolic and diastolic blood pressure ≥ 95^th^ percentile for gender, age and height [2]. Social class was determined using the socioeconomic indices of the parents as described by Oyedeji [20]. Normal weight was defined as BMI- for age between the 5^th^ and 85^th^ percentile, overweight was defined as BMI-for-age between 85^th^ and 95^th^ percentile while obesity was defined as BMI-for-age above 95^th^ percentile [16]. Data were analyzed using SPSS (Statistical Package for Social Science) version 16. Frequency distributions were displayed using tables and charts. The comparison of categorical variables and tests for association was by means of chi-square test (χ^2^). The continuous variables like age and BMI were compared using the students-tests. Statistical significance was set at P-value < 0.05. Multivariate analysis was used to ascertain the association between hypertension and some characteristics of the subjects.

## Results

Of the 1000 subjects recruited, 16 were excluded from analysis based on either incomplete filling of the questionnaire or the presence of significant proteinuria. The remaining 984 adolescents were subjects for this study, and they comprised 470 (47.8%) males and 514 (52.2%) females giving a male: female ratio of 1:1.1. Their ages ranged from 10-19 years. Most of the subjects (47.8%) were aged 13-15 years while only 16.5% were in the age range of 10-12 years. The mean age was 14.6±2.1, 14.7±2.1 and 14.4±2.0 for all, male and female subjects respectively. Most of the subjects 457(46.4%) were of social class III, while only 174 (17.7%) were from the social class I. The remaining 353 (35.8%) were from the middle social class II. Table 1 displays the age and gender distribution as well as the socio-economic status of these adolescents. The mean systolic and diastolic blood pressures were 110.5±10.2mmHg and 71.5±8.5mmHg respectively. Females were found to have higher mean systolic blood pressure (111.4±9.9mmHg) than males (109.5±10.5mmHg), and this difference was statistically significant (t = 2.86, p = 0.004). The mean diastolic BP of females (72.4±8.3mmHg) was significantly higher than that of males (70.6±8.5mmHg), t = 3.32, p = 0.001 (Table 1). Both the mean systolic and diastolic blood pressure increased with the age of the students. Blood pressure distribution among the students showed that 4.3% and 0.5% had systolic hypertension and systolic pre-hypertension respectively. In addition, 4.7% and 5.3% of them have diastolic hypertension and diastolic pre-hypertension respectively. Prevalence of combined systolic and diastolic hypertension and pre-hypertension were 6.3% and 5.0% respectively, as shown in Table 2. There were a higher proportion of females (7.3%) than males (5.4%) with hypertension. More females (5.8%) than males (4.2%) had prehypertension but these were not statistically significant. Most of the adolescents (87.1%) were of normal weight; however, 5.7% and 2.4% were overweight and obese respectively. There were the significantly higher proportion of overweight/obese females (10.7%) than males (5.3%) p-value = 0.001, as shown in Table 3. In addition, there was a higher proportion of overweight/obese adolescents in private schools than in public schools (Table 3). The mean BMI of the girls was higher than the mean BMI of the boys even though the difference was not statistically significant (Table 4). Among the risk factors studied and some other characteristics, only the type of school (private school) (χ^2^ = 7.32, p = 0.01) and BMI (overweight and obesity) (χ^2^ = 42.09, p < 0.001) were found to be significantly associated with hypertension, Table 5. Predictors of hypertension among the students were obesity (O.R = 2.70), school type (O.R = 0.47), sex (O.R = 1.31), age (O.R = 1.23) and family history of hypertension (O.R = 0.79) (Table 6). Adolescents who were obese are thrice at risk of developing of hypertension compared to non-obese adolescents while being in public school reduces the risk of hypertension by 57% (Table 6).

**Table 1:** basic characteristics and mean blood pressures of the adolescents

Age and gender distribution					
Age (years)	Male N=470(%)	Females N=514(%)	Total n=984(%)	*X^2^*	p value
10-12	74(15.7)	88(17.1)	162(16.5)	4.76	0.09
13-15	212(45.1)	259(50.4)	471(47.8)		
16-19	184(39.2)	167(32.5)	351(35.7)		
Total	470(47.8)	514(52.2)	984(100)		
**Socio-economic characteristics**					
Social class	Male N=470(%)	Female N=514(%)	Total n=984(%)		
1	85(18.1)	89(17.3)	174(17.7)		
II	198(42.1)	155(30.2)	353(35.8)		
III	187(39.8)	270(52.5)	457(46.4)		
Total	470(47.8)	520(52.2)	984(100)		
**Mean Blood Pressure**					
Blood pressure	Male mean±SD	Female mean±SD	Total mean±SD	t-test	
Systolic BP	109.5±10.5	111.4±9.9	110.5±10.5	2.86	0.004
Diastolic B	70.6±8.5	72.4±8.3	71.5±8.5	3.32	0.001

**Table 2:** pattern of distribution of blood pressure among the adolescents

Blood Pressure	Male n=470(%)	Female n=514(%)	Total n=984(%)	*X^2^*	p-value
**Systolic BP**					
Normal	453 (96.4)	484 (94.2)	937 (95.2)		
Pre-hypertension	2 (0.4)	3 (0.6)	5 (0.5)	2.69	0.26
Hypertension	15 (3.2)	27 (5.2)	42 (4.3)		
**Diastolic BP**					
Normal	432 (91.9)	454 (88.3)	886 (90.0)		
Pre-hypertension	20 (4.3)	32 (6.2)	52 (5.3)	3.53	0.17
Hypertension	18 (3.8)	28 (5.4)	46 (4.7)		
**Combined DBP and SPB**					
Normal	426 (90.6)	447 (86.9)	873 (88.7)		
Pre-hypertension	19 (4.2)	30 (5.8)	49 (5.0)	4.02	0.18
Hypertension	25 (5.4)	37 (7.3)	62 (6.3)		

**Table 3:** pattern of distribution of BMI among the adolescents

Class	Male n=470(%)	Female n=514(%)	Total n=984(%)	*X^2^*	P value
Underweight	34(7.2)	13(2.5)	47(4.7)	23.06	˂0.001
Normal	411(87.4)	446(87.4)	867(87.1)		
Overweight	14(3.0)	42(8.2)	56(5.6)		
Obese	11(2.9)	13(2.5)	25(2.5)		
Mean BMI±SD	19.42±3.12	20.55±3.26	20.01±3.24		
	Public school n=663(%)	Private school n=321(%)	Total n=984(%)		
Underweight	46(6.9)	1(0.3)	47(4.8)		
Normal	574(86.6)	283(88.2)	857(87.1)	33.21	0.001
Overweight	35(5.3)	21(6.5)	56(5.7)		
Obese	8(1.2)	16(5.0)	24(2.4)		
Mean BMI±SD	19.6±3.0	20.9±3.5	20.0±3.22		

**Table 4:** mean BMI of overweight and obese adolescents by gender

Class	Male Mean±SD	Female Mean±SD	Total Mean±SD	*t-test*	p-value
Overweight(kg/m^2^)	24.79±2.53	25.46±1.34	25.29±1.72	1.27	0.21
Obese (kg/m^2^)	30.54±3.31	29.70±3.98	30.08±3.64	0.56	0.59

**Table 5:** relationship between prehypertension, hypertension and selected characteristics of the adolescents

Characteristic	Normal n=873 (%)	Pre-HTN n=49 (%)	HTN n=62 (%)	*X^2^*	p-value
**Sex**					
Male	427 (48.9)	19 (38.8)	24 (38.7)	3.98	0.14
Female	446 (51.1)	30 (61.2)	38 (61.3)		
**Age (in years)**					
10-12	148 (16.9)	5(10.2)	8 (12.9)		
13-15	419 (48.0)	28 (57.1)	24 (38.7)	6.77	0.15
16-19	306 (35.1)	16 (32.7)	30 (48.4)		
**Type of School**					
Public	576 (66.1)	37 (75.5)	50 (80.6)	7.64	0.02
Private	297 (33.9)	12 (24.5)	12 (19.4)		
**Family History of HPT**					
Yes	171 (19.6)	12 (24.5)	16 (25.8)	1.61	0.45
No	702 (80.4)	37 (75.5)	46 (74.2)		
**Social Class**					
Class 1	152 (17.4)	12 (24.5)	10 (16.1)		
Class 2	321 (36.7)	15 (30.6)	18 (29.1)	4.00	0.41
Class 3	400 (45.9)	22 (42.9)	34 (54.8)		
**BMI**					
Underweight/Normal	811 (92.9)	42 (85.7)	50 (80.6)		
Overweight	50 (5.7)	3 (6.1)	3 (4.8)	46.63	0.001
Obese	12 (1.4)	4 (8.2)	9 (14.6)		

**Table 6:** logistic regression output for characteristics of adolescents associated with hypertension

Risk Factor	O.R	95% CI
School type	0.43	(0.22-0.86)
Sex	1.24	(0.71-2.14)
Age	1.37	(0.91-2.04)
Social Class	1.20	(0.83-1.73)
Family History of HTN	0.79	(0.44-1.53)
Obesity	2.86	(1.76-4.62)

## Discussion

The present study observed a mean prevalence of hypertension of 6.3% (5.4% for boys and 7.3% for girls) among adolescents in Awka South LGA of Anambra state, southeastern Nigeria. This is similar to the report by Mijinyawa *et al* [21] in Kano State Nigeria, who documented a prevalence of 7.2%, (6.7% for boys and 7.7% for girls) in a similar population of 1000 teenagers aged 13-19 years. The finding in this study is also similar to that documented in Calabar by Odey *et al* [22] who observed a prevalence of 6.7% among 375 adolescents. Kehishadi *et al* [23] in Iran documented similar finding where they reported a combined prevalence of 7.7% among students from 23 provinces aged between 6-18 years. Studies elsewhere [24, 25], also documented similar findings. The implication of this is that apparently, healthy adolescents can be hypertensive without knowing and this underscores the need for a regular check of blood pressure among these adolescents. The prevalence of hypertension in this study is higher than 0.1% reported by Oyewole *et al* [26] and 2% documented by Adams-Campbell *et al* [27]. The possible reason for this could be due to differences in methodology. Whereas the present study used the standard mercury sphygmomanometer, Oyewole *et al* [26] used aneroid sphygmomanometer which may give inaccurate blood pressure readings. On the other hand, whereas hypertension was placed at ≥ 95^th^ percentile in the present study, Adam Campbell *et al* [27] fixed hypertension at 140mmHg and 90 mmHg as cut off points for systolic and diastolic hypertension respectively and this can explain the lower prevalence documented in their study. A Similar study in Israel [28] documented a prevalence of 2.1% which is lower than the finding of the present study, but the lower age group (6-9 years) recruited in that study could be an explanation for the lower prevalence observed. Hypertension has been documented to be higher in adolescents than in younger children [29]. Studies [30, 31] in other parts of Nigeria reported prevalence similar to that reported in this study. Ejike *et al* [32] in Kogi State, Nigeria had reported a prevalence of 17.5% (16.9% for boys and 18.0% for girls) which is much higher than the finding of this present study. However, there is a possibility that the electronic sphygmomanometer used by Ejike and co-workers may explain this disparity. Another reason could be that as opposed to present study, which included adolescents, aged 10-19 years, Ejike *et al* [32] studied only those aged 13-18 years who are expected to have higher blood pressures. Papandreou *et al* [33] also documented a similar higher prevalence of 15.1% in a study in Greece. The prevalence of prehypertension observed in the present study is lower than that reported by Ujunwa *et al* [30] and Ejike *et al* [32] but is similar to the findings in other studies [27]. Importantly, the implication is that these adolescents with prehypertension may develop hypertension later in life and this call for close monitoring of these adolescents.

Another important observation in this study is that both mean systolic and diastolic blood pressures were higher in girls than boys, a difference that was statistically significant. A similar observation was made by several studies in the past [26, 29, 31]. Akor *et al* [29] had reasoned that this could be due to the early onset of puberty in girls that could result in a slight increase in both systolic and diastolic BP among these girls when compared to boys. Equally similar to the above finding is the fact that this study also observed a higher prevalence of hypertension (7.3%) among girls than boys (5.4%) did. Other studies on blood pressure and hypertension in the past have reported similar findings [26, 33]. In addition to the reason given above, greater body mass index among girls in the early puberty may also explain this difference. Review of the literature revealed that obesity and overweight are established risk factors for hypertension, [14, 15] and this was confirmed in this study. This study also demonstrated that 5.6% and 2.4% of the subjects were overweight and obese respectively. Overweight and obesity was significantly associated with hypertension, p-value < 0.001. When subjected to logistic regression analysis, obesity was the highest predictor of hypertension. Adolescents who are obese were about three times more likely to develop hypertension when compared to their nonobese counterparts, (OR 2.70; 95% CI = 1.82-4.00). Further analysis revealed that a significantly high proportion of the females (10.7%) were overweight or obese when compared to 5.3% of their male counterparts. Opara *et al*, [34] had earlier documented a similar finding among 983 schoolchildren in Southern Nigeria. Senbanjor and Oshinkoye [35] in Abeokuta South-Western Nigeria documented a significantly higher prevalence of obesity and hypertension among female adolescents when compared to their male counterparts. This is further buttressed by the fact that logistic regression shows that females have at least a 20% higher risk of developing hypertension than males. (OR = 1.31, 95% CI- 0.86-1.99). Studies in Sudan [36] and India [37] also observed findings similar to that documented in the present study. Although the reason for the high prevalence of overweight and obesity among adolescent girls may not be clearly understood, one known fact is the hormonal interplay that heralds puberty occurs earlier in females and encourages much deposit of fat in females. This could account for the higher BMI that may result in a higher prevalence of hypertension among these females. Social class and family history of hypertension are two other important factors that may influence the development of adolescent hypertension. This study has not shown any significant association between social class, family history, and hypertension. As in the present study, Zeena *et al* [36] in a study in Sudan demonstrated that obesity, but not a family history of hypertension was associated with hypertension among 304 children that participated in their study. Unlike the present study, Nichols *et al* [38] documented a positive correlation between family history and adolescent hypertension where they noted that a third of all the adolescents with hypertension had parents, siblings, or grandparents diagnosed or been treated for hypertension. However, there is no immediate explanation for this disparity. Logistic regression of the possible predictors of hypertension revealed that being in public school reduces the risk of hypertension by 57%. This could be explained in part by the fact that obesity, which is a risk factor for hypertension in this study, is significantly lower among children in the public school as opposed to their counterparts in the private school who are likely to come from the elite class. Children of the elite class are more likely to overfeed and indulge in habits like watching movies for a long time (inactivity) which leads to obesity.

## Conclusion

Hypertension and prehypertension exist among secondary school adolescents in Awka South Local government area of Anambra state, at a prevalence of 6.3% (5.4% for males and 7.3% for females). Overweight and obese females have higher blood pressures than the rest. To detect early and institute intervention for adolescents with abnormally high blood pressure, there is a need for periodic blood pressure monitoring as part of the school health programme.

### What is known about this topic

- There is a need for early detection and treatment of primary hypertension in adolescents;
- Despite the need, data on the prevalence in southeastern Nigeria is scarce.

### What this study adds

- The observed prevalence of primary hypertension (6.3%) and prehypertension (5%) among secondary school adolescents in Awka, Anambra state, advocates for routine blood pressure check;
- In these adolescents, attending private schools, female sex and being overweight or obese are predisposing factors to having higher blood pressures.

## Competing interests

The authors declare no competing interests.
